# Advanced Inorganic Nitride Nanomaterials for Renewable Energy: A Mini Review of Synthesis Methods

**DOI:** 10.3389/fchem.2021.638216

**Published:** 2021-07-09

**Authors:** Yin Ma, Lijun Xiong, Yao Lu, Wenqiang Zhu, Haihong Zhao, Yahui Yang, Liqiu Mao, Lishan Yang

**Affiliations:** Key Laboratory of Chemical Biology & Traditional Chinese Medicine Research (Ministry of Education of China), National and Local Joint Engineering Laboratory for New Petrochemical Materials and Fine Utilization of Resources, Key Laboratory of the Assembly and Application of Organic Functional Molecules of Hunan Province, Hunan Normal University, Changsha, China

**Keywords:** nitride nanomaterials, controllable synthesis, energy storage, two-dimensional materials, defects

## Abstract

Inorganic nitride nanomaterials have attracted widespread attention for applications in renewable energy due to novel electrochemical activities and high chemical stabilities. For different renewable energy applications, there are many possibilities and uncertainties about the optimal nitride phases and nanostructures, which further promotes the exploration of controllable preparation of nitride nanomaterials. Moreover, unlike conventional nitrides with bulk or ceramic structures, the synthesis of nitride nanomaterials needs more accurate control to guarantee the target nanostructure along with the phase purity, which make the whole synthesis still a challenge to achieve. In this mini review, we mainly summarize the synthesis methods for inorganic nitride nanomaterials, including chemistry vapor deposition, self-propagation high-temperature synthesis, solid state metathesis reactions, solvothermal synthesis, *etc*. From the perspective of nanostructure, several novel nitrides, with nanostructures like nanoporous, two-dimensional, defects, ternary structures, and quantum dots, are showing unique properties and getting extensive attentions, recently. Prospects of future research in design and synthesis of functional inorganic nitrides are also discussed.

## Introduction

**FIGURE 1:**
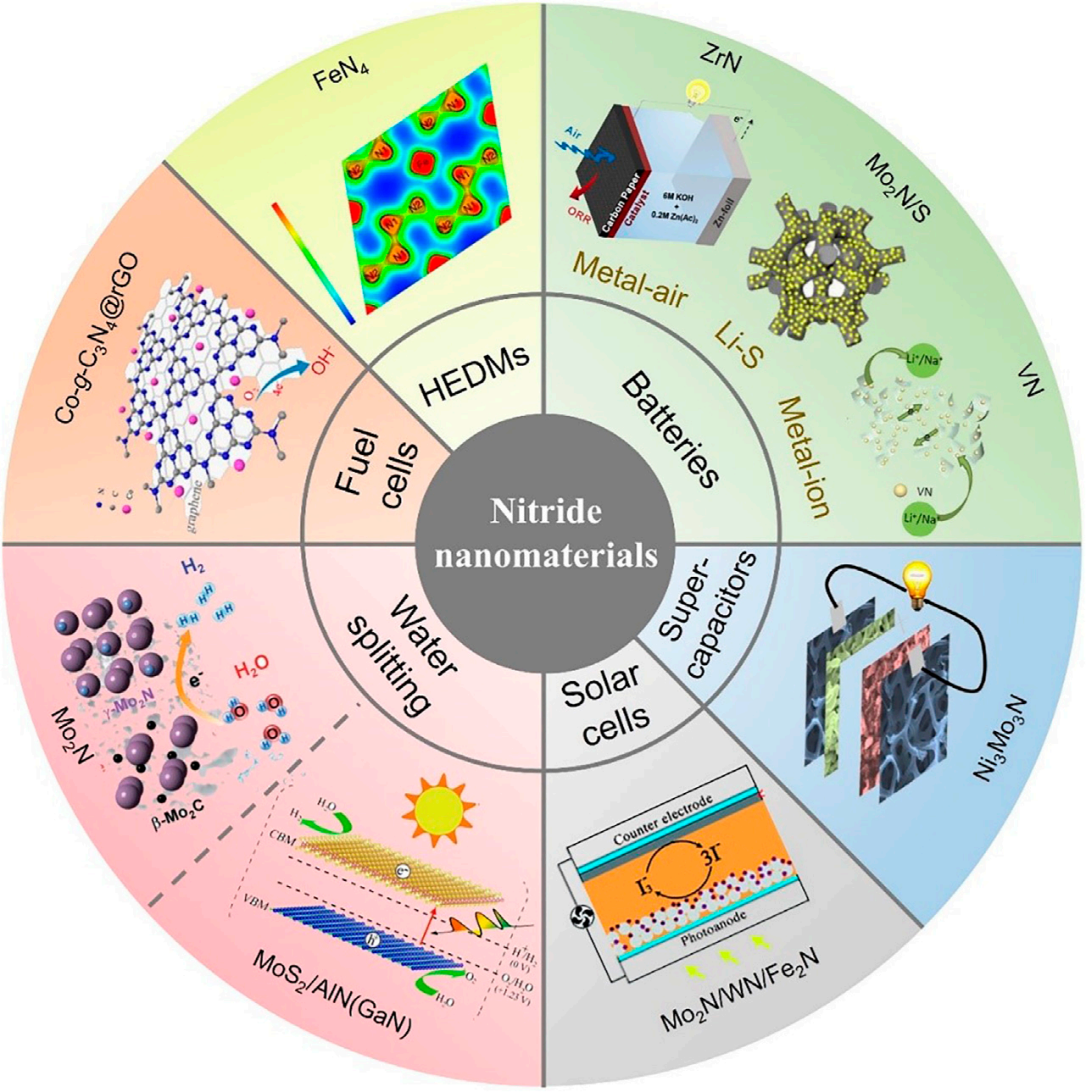
Overview of inorganic nitride nanomaterials for renewable energy applications, including ZrN for zinc-air batteries, mesoporpus Mo_2_N/S for Li-S batteries, hybrid 2D–0D Graphene–VN quantum dots for lithium and sodium ion batteries, Ni_3_Mo_3_N_3_ nanorods for supercapacitors, Mo_2_N or WN or Fe_2_N for solar cells solar cells, heterostructural MoS_2_/AlN(GaN) for photocatalytic water splitting, 3D porous Mo_2_N for electrocatalytic hydrogen evolution, 2D Co-g-C_3_N_4_ bulk for fuel cells and FeN_4_ as high energy density materials. The inserted graphics are adapted with permission from Li et al. (2011), Chen et al. (2013), Liu and Zhang (2013), Liao et al. (2014), Wang et al. (2016), Bykov et al. (2018), Jiang et al. (2018), Kumar et al. (2020), Yuan et al. (2020).

Increasing demands for renewable energy have stimulated the ever-growing developments in the generation of novel energy storage and conversion technologies (Chu et al., 2016; Chen et al., 2020; Vakulchuk et al., 2020). Various inorganic nitride nanomaterials have been designed, for not only conventional applications (E.g., lubricants, cutting materials, semiconductors, luminescent materials, *etc.*), which also attract increasing interests in renewable energy (Figure 1) and (Supplementary Table S1), such as, electrochemical hydrogen evolution reaction (WN, ZrN) (Wang et al., 2015; Zhong et al., 2016), rechargeable batteries (VN, Mo_2_N) (Zhang et al., 2019; Tapia-Ruiz et al., 2020), supercapacitors (TiN, NbN, Mn_2_N_3_, Ni_3_N) (Gray et al., 2017; Ghosh et al., 2018), solar cells (Ni_3_N) (Balogun et al., 2015; Prasad et al., 2018), and fuel cells (ZrN, CoN) (Dong et al., 2013; Yuan et al., 2020). In terms of element composition, functional nitrides mainly include transition metal nitrides and non-metal nitrides. For transition metal nitride nanomaterials, the bonding between metal atoms and nitrogen atoms will change/shrink the *d*-band structure of host metals, which fundamentally change the activity of catalytic sites and support transition metal nitrides to gain electronic structure and electrocatalytic activity like noble metals. Besides, unique electronic characteristics and strong metal-nitrogen bonds make the nitride anion (N^3−^) difficult to be substituted, further leading to transition metal nitride nanomaterials show properties like hardness, mechanical stiffness, and high chemical stability. For non-metal nitride nanomaterials (including BN, C_3_N_4_ and C_*x*_N), typical N configurations (like quaternary N, pyridinic N and pyrrolic N) are formed within the boron or carbon skeleton, of which quaternary N and pyridinic N could show activities for hydrogen evolution reaction (HER) and oxygen evolution reaction (OER) in water splitting, or oxygen reduction reaction (ORR) in proton exchange membrane fuel cells (PEMFCs) (Liu et al., 2015; Niu and Yang, 2018). Moreover, physical and chemical modifications could furtherly support BN obtain tunable band gap or high porosity, which enable those modified BN nanomaterials with various energy storage applications (Han et al., 2020). Above all, inorganic nitride nanomaterials show various possibilities for renewable energy applications.

Over the last decades, the applications of functional nitrides grow with the development of new synthetic routes and analysis technologies (Horvath-Bordon et al., 2006; Mazumder et al., 2008; Zhong et al., 2016; Hong et al., 2020; Li Y. et al., 2020; Wu et al., 2020). Although the fabrication of inorganic nitrides has made significant progress, and many new nitride phases or new nanomaterials or new applications have been produced, the synthesis process for nitrides is always complex and has a large thermodynamic barrier to overcome (945 kJ mol^−1^ for the formation/break of N≡N bonds, compared to 498 kJ mol^−1^ for that of O=O bonds) (Balogun et al., 2015). Moreover, for the synthesis of nitride nanomaterials, it is a much more difficult challenge to realize the multi-parameter control of its structure, morphology, size, doping, and defects. In this review, we summarized the mostly used synthesis methods of inorganic nitride bulk-materials or nanomaterials and their corresponding development trends. Some nitride nanomaterials with various morphologies have been presented along with their fabrication operations and the applications as renewable energy. Besides, several unique nanostructures are specially discussed: nanoporous, defects, two-dimensional (2D), ternary nitrides, and quantum dots. Lastly, we conclude with the challenges and chances of precise functionalized inorganic nitrides.

## Synthesis Methods for Nitrides

**FIGURE 2:**
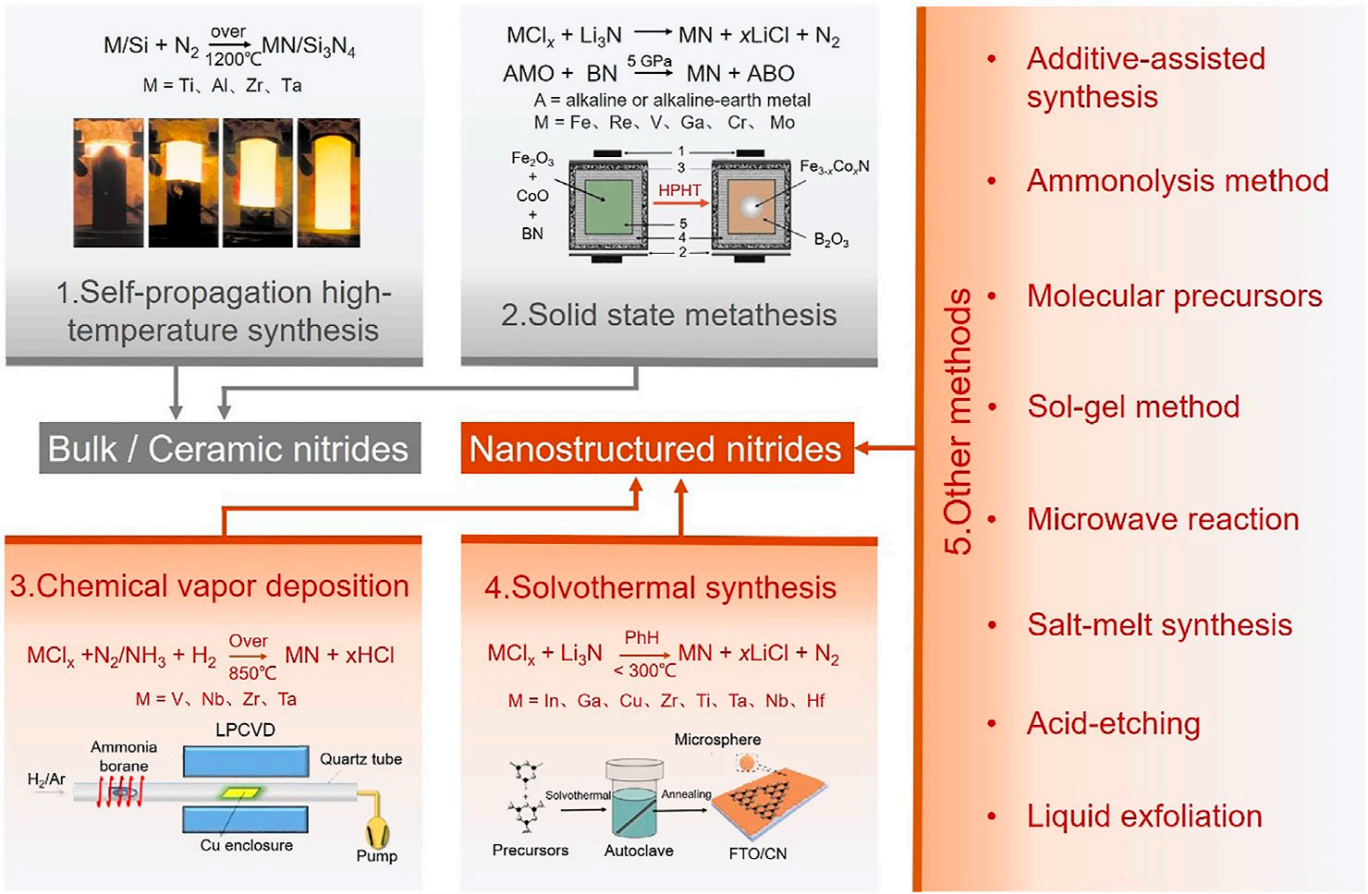
Schematic illustration of various synthetic methods to inorganic nitrides, where methods 1 and 2 are mostly used to produce bulk or ceramic nitride materials (marked in grey), and methods 3, 4 and 5 are mostly used to prepare various nitride nanomaterials (marked in red). Method 1 of self-propagation high-temperature synthesis reproduced with the permission from Amiour et al. (2016). Method 2 of solid state metathesis reactions reproduced with the permission from Lei and Zhang (2018). Method 3 of chemistry vapor deposition reproduced with the permission from Song et al. (2015). Method 4 of solvothermal synthesis reproduced with the permission from Xie et al. (2016). Recently, some new synthesis methods for nitride nanomaterials are derived from the above four methods, including additive-assisted synthesis, ammonolysis, molecular precursors method, sol-gel, microwave reaction, salt-melt synthesis, acid-etching and liquid exfoliation.

The electrochemical activity and chemical stability of nitride nanomaterials are highly dependent on the structures of related nitride nanomaterials. Therefore, the design and fabrication of high-quality nitride nanomaterials is the initial and key steps to realize their applications for renewable energy. Traditionally, nitrides are generally synthesized under high temperatures (800–1500°C) or high pressure, which makes it easier to obtain nitride materials with bulk or ceramic structures. In order to obtain nanostructured nitrides, active precursors or halide raw-materials (E.g., TiAlN_*x*_, SiCl_4_, BBr_3_) would be selected, and additives or active intermediates (E.g., S, I_2_, H_2_O, NaBH_4_, NaN_3_) are always introduced. (Gillan and Kaner, 1996; Spatz et al., 1999; Sardar et al., 2005; Mazumder and Hector, 2009; Yang et al., 2010; Chen et al., 2012; Xu et al., 2012; Zhou et al., 2017). In this work, classic synthesis methods of nitrides are summarized into a few categories (Figure 2).

### Self-Propagation High-Temperature Synthesis

The SHS method (or named as combustion synthesis) is used to describe a process in which initial reagents spontaneously transform into products due to the exothermic heat of reaction, once ignited (Subrahmanyam and Vijayakumar, 1992). Due to the fast-release of heat from the exothermic reaction, the time to meet the combustion temperature is short and the conversion rate to products is high (Merzhanov, 2004). For example, a divalent europium-doped nitride phosphors Ca_1-*x*_Eu_*x*_AlSiN_3_ (x = 0–0.2) were prepared by heating Ca_1-*x*_Eu_*x*_AlSi alloy powders under continuous N_2_ flow at 1050°C (Piao et al., 2007). Wang’ groups reported the selective synthesis of four types of BN nanotubes by using a porous precursor through the SHS method (Wang et al., 2011). SHS is a practical method for large-scale preparation of nitride powders, but it is difficult to achieve the controllable preparation of nitride nanomaterials.

### Solid State Metathesis Reactions

Solid state metathesis has been widely used to produce bulk or nano-sized metal nitrides. The core design of SSM is the replacement reactions between metal halides and alkali (or alkaline earth) nitrides (E.g., Li_3_N and NaN_3_), yielding corresponding metal nitrides (Wiley and Kaner, 1992; Zhu et al., 2011; Karaballi et al., 2019), as described in Eq. 1. When producing low thermal stability nitrides (E.g., GaN, InN, Zn_3_N_2_, Ta_3_N_5_), the synthesis temperature need to be controlled (Gillan and Kaner, 1994).

$$\mathrm{MC}l_{x}\mathrm{+x}\mathrm{/3 L}i_{3}N\to \mathrm{MN}+x\mathrm{LiCl}+\mathrm{(x}\mathrm{-3)/6}N_{2}$$ (1)

A high-pressure solid-state metathesis (HPSSM) reaction was reported between boron nitride (BN) and ternary metal oxide A_*x*_M_*y*_O_*z*_ (A = alkaline or alkaline-earth metal and M = main group or transition metal), as presented in Eq. 2. Then, a series of well crystallized metal nitrides (Fe_3_N, Re_3_N, VN, GaN, CrN, and W_*x*_N) were produced (Lei et al., 2013). In subsequent reports, high pressure was confirmed in SSM to benefit the contact diffusion and ion-exchange of the solid reactions (Lei and Zhang, 2018). Well-crystallized W_*x*_N nanomaterials were synthesized *via* the HPSSM, where solid-state ion exchanges and a nitrogen pressure of 5 GPa were involved (Wang et al., 2012). According to DFT calculations of thermodynamic investigations, high pressure could reduce the reaction enthalpy △H.

$$A_{x}M_{y}O_{z}+\left(\mathrm{2z-a}x\right)\mathrm{/3}\mathrm{BN}\to y\mathrm{/w}M_{w}N\mathrm{+ 0.5}\left[\left(\mathrm{2z-a}\mathrm{x/3-}\mathrm{y/w}\right)\right]N_{2}+A_{x}B_{\mathrm{(2z-a}\mathrm{x)/3}}O_{z}$$ (2)

Moreover, kinetically controlled SSM reactions provide an avenue toward the synthesis of nitride materials (Rognerud et al., 2019). For example, Mn_3_N_2_ micro-/nano-crystals were prepared by a kinetically controlled SSM reactions. The further developments of SSM for inorganic nitride nanomaterials would be focused on lower pressure and the kinetic control, where slightly mild and mild kinetics are helpful for the controllable preparation of nanostructures in a exothermic reaction.

### Chemistry Vapor Deposition

Chemistry Vapor Deposition (CVD) method is widely favored by chemists, physicists, and material scientists, by which nitride nanomaterials (mainly thin layers, geometric nano-sheets, nano-column, or nano-flowers, *etc.*) were fabricated by heating volatile metal halides under flow gas (E.g., N_2_+H_2_ or NH_3_+H_2_, *etc.*) at high temperatures (above 850°C) (Fix et al., 1993; Zerr et al., 2003), as illustrated in Eq. 3. The replacement of metal chlorides by metal-organic salts could reduce the reaction temperatures to 200–450°C (Fix et al., 1996).

$$\mathrm{MC}l_{x}+N_{2}\mathrm{/N}H_{3}+H_{2}\mathrm{\to MN}+x\mathrm{HCl}$$ (3)

Recent reports focused on the kinetic control and the nano-structural evolutions during the CVD processes. For CVD method, the substrate places an important role for the growth of nitride crystals in terms of the phase and the crystal morphology. In a vapor-liquid-solid case, high-quality GaN nanowires were synthesized *via* metal-initiated metalorganic CVD (Kuykendall et al., 2003). For another example, high-quality *h*-BN mono-layer was prepared by using a folded Cu-foil enclosure (Song et al., 2015). Smooth Cu surface effectively reduced the precursor feeding rate, leading to a drastic decrease in the nucleation density. Density functional theory (DFT) calculations demonstrated the crystallographic orientation of the Cu substrate strongly correlated with the orientation of *h*-BN. Similar phenomena appear in the silicon-assisted growth of centimeter-scale MoSi_2_N_4_ monolayers (Hong et al., 2020). It can be predicted that more and more nitrides of the 2D thin-layered family will be synthesized by CVD.

### Solvothermal Synthesis

Solvothermal reactions are carried out in organic solvents within an autoclave under a certain temperature and autogenous pressure, where the solvents dissipate the enthalpy of the exothermic metathetical reactions and reduce the diffusion barriers between reactants (Demazeau, 2007). Thus, the solvothermal reactions could be occurred at a relative low temperature and provide better control over the size and morphology of the particles (compared to solid state reactions like SHS or SSM).

In early reports, solvothermal synthesis of nitride nanomaterials mainly explored the regulation of metastable phases, different solvents, and nano morphologies. For example, the reaction between GaCl_3_ and Li_3_N in benzene could yield hexagonal/rocksalt mixed-phase GaN nano-crystallites at 280°C (Xie et al., 1996). InN could be synthesized by reactions between InCl_3_ and Li_3_N in xylene at 250°C (Demazeau et al., 2002). Later, CrN, ZrN, TiN and NbN were obtained from reactions of chlorides with NaN_3_ in benzene (Mazumder and Hector, 2009). Similarly, solvothermal methods have been applied to synthesize group 5, 6 metal nitrides in different organic solvents (Mazumder et al., 2008; Bugaris and Ibers, 2010). In recent years, many nitride nanomaterials were fabricated and studied for various renewable energy applications. A Cu_3_N nanocubes were fabricated in a mixed solvent of ODA and OAm, which exhibited room-temperature ferromagnetism and excellent electrocatalytic activity for ORR and nitrobenzene reduction (Xi et al., 2014). Oxygen-doped *g*-C_3_N_4_ hollow nanospheres were synthesized from acetonitrile solvothermal method at 180°C, which showed remarkably photocatalytic activity for environment pollutant purification and splitting water for HER (Wang et al., 2017). Compared to other methods, solvothermal synthesis is energy-efficient and easy-control. Besides, the reaction is carried out in a closed system, which can effectively prevent the oxidation and uneven growth of nanocrystals, then becoming popular for the synthesis of nitride nanostructures.

### Other Synthetic Methods

Besides four traditional synthetic methods, we summarized some other novel methods for inorganic nitride nanomaterials. Generally, thermodynamics driven nanocrystals are easily obtained from additive assisted synthesis; ammonolysis, molecular precursors, sol-gel method and salt-melt synthesis are based on the precursor design with similar principles; microwave synthesis uses the high energy of microwaves for a quickly synthesis; spin-steaming, acid-etching, liquid-exploration, MOFs-assisted synthesis are designed to prepare nitrides with certain characteristic nanostructures (Figure 2).

### Additive-Assisted Synthesis

Inspired by SSM and solvothermal reactions, the key to efficient synthesis of nitride crystals is to select thermodynamically spontaneous reactions and raw materials with high activity. In our previous works, a series of nitride (E.g., TiN, BN, AlN, MgSiN_2_, VN) nanocrystals were prepared from metal oxides with the assistant pre-reactions of metallic Mg (or Na) with H_2_O (or S) at a low temperature. In detail, TiN, ZrN, BN, and AlN were prepared by using the corresponding elements (Ti, Zr, B, Al), NaN_3_ and sulfur as starting materials at 250°C (Yang et al., 2010). The exothermic reaction of additives (Na and S) was taken to prepare TiN, BN, AlN, MgSiN_2_, VN nanocrystals, and the raw materials were metal oxides and NaN_3_. Due to the additives, the reaction temperature was lowered at 150°C (Chen et al., 2012). And a metal-hydrolysis-assisted synthesis (MHAS) strategy was used to synthesis nitrides (VN, AlN, CrN, MgSiN_2_ *etc.*) from related metal oxides in a low temperature range (120–180°C) (Zhou et al., 2017). Additive-assisted synthesis is very efficient, but the controllability of nanostructures needs further optimization.

### Ammonolysis

Temperature programmed ammonolysis was developed, which involves heating the metal oxide in flowing NH_3_ and slowly raising the temperature (Hausler and Schnick, 2018). Crystallographic orientations, particle size, and geometry are retained from the reactant solid. For example, S. Imran U. reported an ammonolysis reaction of MoCl_5_ and [Mo (NMe_2_)_4_] to form two different polymeric precursors, where the cubic Mo_2_N from Mo(NMe_2_)_4_ exhibited lattice distortions and significant redox properties as the supercapacitor electrode, and hexagonal Mo_2_N (observed from the MoCl_5_) with higher surface areas a had a high capacitance of 275 F g^−1^ (Shah et al., 2014). Also, binary Ni_2_Mo_3_N nanorods were synthesized similarly, which showed excellent electrochemical performance for supercapacitors with a specific capacity of 264 F g^−1^ at 0.5 A g^−1^ and 81.4% capacity retention after 1000 cycles (Kumar et al., 2020). Moreover, through this Ammonolysis method, ultrathin 2D GaN (∼2 nm thick) and InN (∼1.3 nm thick) were synthesized from liquid metal (Syed et al., 2019).

### Molecular Precursors Method

In this way, nitrogenous precursors or intermediate products were firstly synthesized, then the precursors/intermediates would follow by a low-temperature decomposition and a high-temperature calcination, through which the size and morphology of the nanocrystals can be controlled by adjusting the temperature and the heating time. For example, nitride (ZrN, TiN, NbN) nanomaterials are pyrolyzed by reactions of Zr^4+^, Ti^4+^ and Nb^5+^ dialkylamines with liquid ammonia to produce insoluble precipitates (Brown and Maya, 1988). Also, group 13 nitrides could be obtained by refluxing urea derivatized AlCl_3_, InCl_3_ and GaCl_3_ precursors in TOA, then hexagonal GaN and cubic AlN, InN are generated (Sardar et al., 2005). It is worth learning that Lee used a urea precursor to prepare mesoporous carbon nitride nanostructures with silica nanospheres as a hard template (Lee et al., 2012).

### Sol-Gel Processing

Sol-gel methods are wildly used for producing nitride nanomaterials, such as nano-powders or nanoporous structures (Giordano and Antonietti, 2011). During the whole process, ions-organics molecular precursors are formed in solution, then heated the reacted to yield a gel, and finally fired to the target nitrides. For example, TiN nanoparticles (NPs) were coated on carbon fibers *via* a sol-gel method based on self-condensation of titanium alkyl amide species. In this work, the capacity of the coated fibers was increased with significant redox capacitance contributions (Zhang et al., 2018). In another example, ZrN NPs have been prepared by heating the ZrCl_4_-Urea sol-gel under an argon flow, which showed better ORR activity than commercial Pt/C catalysts in an alkaline environment (Yuan et al., 2020). In general, the size, morphology, and porosity of the sol-gel prepared nitrides can be controlled by adjusting the organic content, reaction conditions and templates or supports.

### Salt-Melt Synthesis

Salt-melt synthesis (SMS) is a special synthesis method in which the reaction happen in the liquid molten salts and products will be obtained by washing the cooled salts when the reaction is completed. There are two main advantages of SMS. For the first, SMS can lower the synthesis temperature and shorten the reaction time. For example, by using the SMS, the size and morphology of the nitride nanoparticles can be well controlled even under lower synthesis temperature (Liu et al., 2013). And secondly, SMS is a convenient and efficient way to fabricate 2D films or 3D porous nanostructures. For example, 3D porous N-doped graphene (HNG) was synthesized by SMS method, which display super bifunctional catalytic activity toward both ORR and OER (Cui et al., 2018). Also, a family of 2D layered transition metal nitrides (TMNs, such as MoN_1.2_, WN_1.5_, and Mo_0.7_W_0.3_N_1.2_ *etc.*) could be produced under an atmospheric pressure and showed superior performance in HER (Jin et al., 2020). Lastly, the choice of salts is important in SMS, especially for the large-scale (over decagram) synthesis with High purity requirements.

### Microwave Synthesis

Microwave synthesis is a modern synthesis technique under the condition of microwave, which has advantages of rapid heating, homogeneity, and selectivity (Tompsett et al., 2006). Since microwaves can penetrate deep into the substance, it only takes one-tenth to one-hundredth of the time of conventional methods to complete the entire heating process. The size of the nitride products is small and its distribution is uniform. For example, Graphitic carbon nitride (*g*-C_3_N_4_) sub-microspheres were prepared *via* a facile microwave synthesis through polymerization reaction between C_3_N_3_Cl_3_ and NaN_3_ in acetonitrile solvent (Dai et al., 2013). Guo et al. produced a *g*-C_3_N_4_ material after optimizing the microwave reaction time which can effectively generate H_2_ under visible-light irradiation (Guo et al., 2016). In this work, the highest H_2_ evolution rate achieved was 40.5 mmol h^−1^, which was two times higher than that of *g*-C_3_N_4_ products prepared by other methods.

In addition to the above methods, two novel synthesis methods of nitride materials have been developed recently. One is acid-etching, E.g., multilayered Ti_2_N sheets were achieved by the selective etching of ternary layered Ti_2_AlN with a mixture of potassium fluoride and hydrochloric acid, which showed excellent SERs efficiency (Soundiraraju and George, 2017). Another method named as liquid exfoliation, E.g., atomically-thin MoN nanosheets were obtained by liquid exfoliation of MoN precursors in N methyl-pyrrolidone (NMP) after ultrasound treatment for about 8 h, which had wonderful HER performance (Xie et al., 2014). It is still quite difficult to prepare high-quality nitrides by traditional methods, because the obtained nitrides always contain a certain number of nitrogen vacancies, and the miscibility of products is common. These problems are the same important as the control of nanostructures. In addition, it is undeniable that the high-pressure effect can effectively improve the high temperature thermal stability of nitrides and inhibit the dissipation of nitrogen atoms. In conclusion, the selection of suitable precursors and substrates, and the control of high-pressure reaction path are still the keys for the synthesis of nitride nanomaterials with high-purity.

## Controllable Preparation of Specific Nanostructures

Due to the crystalline variety, the obtained morphologies of various nitride crystals are diverse, including nanopyramids, core-shell, nanoporous sheets, nanoflowers, quantum dots, nanotubes, nanoflowers, nanocubes, nanorods, etc. (Supplementary Figure S1) Driven by growth dynamics, BN or C_*x*_N with layered structures are generally to form flake-, tubular-, film-, or porous-like nanostructures, while metal nitrides with cubic or tetragonal structures are preferred to form particles, rods, wires, or dendrites. In this section, nitrides with several specific nanostructures (E.g., nanoporous, 2D, defects, ternary, and quantum dots) are specially illustrated in detail (Supplementary Figure S2).

### Porous Nitrides

Nanostructured nitrides with a large surface area and pore volume are much expected because the confined-space effects inside the pore-structures would improve their chemical and physical performance to meet the demands of electrode/catalysis materials for renewable energy (Wan et al., 2006) As we known, the soft-templating approach is effective and most wildly used to fabricate ordered micro-/meso-porous metal oxides, metal sulfides, metal phosphates, silicates, and zeolites. However, it is difficult to apply soft-templating approach for the preparation of porous metal nitrides due to the lack of proper raw materials for the sol-gel or molecular precursor processes (Yang and Huang, 2005). Present studies mainly adopted the hard-templating method for the development of nitride porous nanostructures (Wan and Zhao, 2007). For example, GaN, VN, and TiN nanoparticles have been synthesized inside the confinement space of porous templet materials (E.g., ordered mesoporous: MCM-41 and SBA-15; disordered mesoporous C_*x*_N), and nitride products with porous composites or isolated nanoparticles would be left after the removal of mesoporous hard templates (Yang and Zhao, 2005; Kogiso et al., 2007; Uedono et al., 2007). Also, self-supported ordered mesoporous metal nitrides (CrN) have been reported (Shi et al., 2008)

### Nitrides With Defects

Nitrides with defects have shown many unique functions, where defects in nitrides are detrimental to the electrode or catalytic reactions by working as a recombination center of charge carriers (Van de Walle and Neugebauer, 2004; Wang et al., 2018; Takamura et al., 2020). Reasonable control of defects in nitrides is the key to the excellent performance of these nitrides. Li’ groups proposed a method to control the defects of carbon nitrates, which can effectively influence the defects of PHI-type CN nanocrystals (Li H. et al., 2020). Defects in *h*-BN are promising single-electron emitter materials with the advantages of high brightness and stability under room temperatures (Abidi et al., 2019). In the process of molten-salt polymerization with melamine as the precursor, KOH were added to realize the nitrogen defect regulation of carbon nitride. For the case of CVD, backside boron gettering technology is used to control the diffusion of B into Cu, thus controlling the defect growth in *h*-BN (Abidi et al., 2019). In addition, ion implantation can also be used to introduce the boron-vacancy, like ion beams (E.g. nitrogen, xenon, and argon) (Kianinia et al., 2020).

### 2D Materials

2D nitride materials (E.g., graphene, BN, MoN, and MXene, *etc*.) have received widespread attentions, because the electron migration and heat diffusion are confined to nano-sized two-dimensional planes, further leading to excellent electrical, optical, and mechanical properties (Naguib et al., 2014; Anasori et al., 2017). For example, 2D transition metal nitrides (MXene) have great application prospects in energy storage, like lithium and sodium ion batteries (Urbankowski et al., 2017). And 2D BN nanomaterials show highly stable structure and unique photoelectric characteristics as graphene, which are mainly prepared by exfoliation, CVD, and vapor phase epitaxy (Zhang et al., 2017). MoN nanosheets was prepared *via* a liquid exfoliation of annealed and can be used as effective HER electrocatalyst (Xie et al., 2014). Nitride-based MXene was prepared by etching the A atomic layer of the layered precursor MAX phase by HF. For example, 2D Ti_4_N_3_ is obtained by etching the Al atomic layer in Ti_4_AlN_3_ with molten fluoride under a condition of high temperatures (Soundiraraju and George, 2017). Many 2D nitride nanomaterials show great application potentials in energy storage (Xiao et al., 2019).

### Ternary Nitrides

Ternary nitrides are usually formed by combining alkali metals or alkaline earth metals and transition metals with nitrogen elements. In such combinations, the cations can stabilize the high oxidation state N^3−^ anions (Balbarin et al., 1996). No more than 400 kinds of ternary metal nitrides have been synthesized (Sun et al., 2019). Compared with binary nitrides, ternary nitrides exhibit more extensive functions and adjustable plasmonic performance (Ran et al., 2019). As the ammonia generated by ammonolysis can damage the test instrument during characterization process. The common synthetic methods for synthesizing ternary nitrides are: HPSSM, solvothermal, ammonolysis, sol-gel, *etc*. For example, the Ti_0.5_Cr_0.5_N was synthesized by ammonizing a solid metal oxide (ZnMO, M = Cr, Ti). By using the HPHT method, a series of ternary materials (Mo_0.23_Nb_0.77_N_0.66_ and Mo_0.66_Nb_0.34_N_0.60_) were synthesized (Tareen et al., 2019). And a template reaction method was reported to prepare continuously adjustable metal composition ternary nitride nanoparticles (Al-Ga-N and Ti-V-N) by using mesoporous graphite carbon nitride as the nanoreactor and reactant (Fischer et al., 2008). It seems that sol-gel method is preferred for the synthesis of ternary nitrides.

### Carbon Nitride Quantum Dots

Graphite phase carbon nitride (*g*-C_3_N_4_) with few nanometers can be named as *g*-C_3_N_4_ quantum dots (*g*-CNQDs), and they always exhibit enhanced photo-absorption and photo-response comparing with the bulk *g*-C_3_N_4_ materials due to the quantum confinement effects (Albolkany et al., 2020). Because of these properties, *g*-CNQDs showed wide range of applications that include photocatalysis, fluorescence probes, drug delivery, bioimaging, and security Ink, etc. (Zhang et al., 2014; Yin et al., 2017; Dong et al., 2018; Patir and Gogoi, 2018) Basically, *g*-CNQDs could be prepared utilizing two methods: the top-to-down way, or the bottom-to-up way. The top-to-down strategy might involve the fragmentation of the bulk *g*-C_3_N_4_ by chemical exfoliation, thermal treatment or ultrasonication into *g*-CNQDs (Song et al., 2016); and the bottom-to-up strategy is usually based on thermal treatment of nitrogen-rich organic precursors into well dispersed *g*-CNQDs (Barman and Sadhukhan, 2012; Tang et al., 2014). It can be expected that *g*-CNQDs will receive growing attentions in the field of functional nanomaterials.

## Conclusions and Outlook

Inorganic nitride nanomaterials have excellent corrosion resistance, special semiconductor structures, and abundant active sites, which make inorganic nitride nanomaterials show great potentials for various applications in renewable energy. In this review, we summarize several important synthesis methods of nitrides (E.g., SHS, SSM, CVD, solvothermal, additive-assisted synthesis, sol-gel, molecular precursor, *etc.*) and some unique structures (E.g., porous, 2D, defects, ternary nitrides, and C_*x*_N quantum dots). Also, we briefly analyzed the advantages and disadvantages of some synthesis methods in the preparation of nitride nanomaterials, and discussed the relationship between nanostructures and characteristics for renewable energy applications.

Based on the above analysis, we think that the synthesis of nitride nanomaterials for renewable energy will present the following three aspects in the future research. (Ⅰ) As thermally stable nitrides are always fabricated *via* above routes from related compounds, it’s still a big challenge to avoid trace impurities and build perfect triple bonds in dinitrogen during the nitride synthesis. Thus, some researchers will focus more on the surface nitridation, surficial atomic composition, and surface activity of functional nitride nanomaterials. (Ⅱ) The synthesis and electrode process of nitride nanomaterials are complex heterogeneous reactions, most of which are reversible or irreversible redox reactions. Therefore, with the help of cryo-electron microscopy, thermal ablation corrected technology, *in-situ* characterization technologies, we can more closely understand the growth truth of nitride nanomaterials and the origins of their activity/failures during the applications. (Ⅲ) The physical and chemical properties of nitride materials are mainly affected by their crystal structures and electronic structures. And DFT calculations may greatly support the design and preparation of complex nitrides under more precise conditions with higher degree of controllability.
