# Supplementary material for: Advanced Inorganic Nitride Nanomaterials for Renewable Energy: A Mini Review of Synthesis Methods
Source: Front Chem. 2021 Jul 9;9:638216. doi: 10.3389/fchem.2021.638216 (PMC8299337; doi:10.3389/fchem.2021.638216)
Supplement: Supplementary file 1 [file DataSheet1.docx]

**Supporting Information**

**Advanced** **inorganic nitride nanomaterials for** **renewable energy: a mini review of synthesis methods**

Yin Ma ^a^, Lijun Xiong ^a^, Yao Lu ^a^, Wenqiang Zhu ^a^, Haihong Zhao ^a,^*, Yahui Yang ^a^, Liqiu Mao ^a^, Lishan Yang ^a,^*

^a^ Key Laboratory of Chemical Biology & Traditional Chinese Medicine Research (Ministry of Education of China), National and Local Joint Engineering Laboratory for New Petrochemical Materials and Fine Utilization of Resources, Key Laboratory of the Assembly and Application of Organic Functional Molecules of Hunan Province, Hunan Normal University, Changsha, Hunan 410081, P.R. China

*** Correspondence:**

zhh_1998@163.com (H.H.Z.)

lsyang.chemistry@gmail.com (L.S.Y.)

Tel./Fax.: +86 731 88872531

Table 1 Synthesis method, condition, morphology, size and application of some nitrides

| Products | Synthetic method | Conditions | | | | Application | Ref. |
| --- | --- | --- | --- | --- | --- | --- | --- |
|  |  | **Temperature** | **Pressure** | **Morphology** | **Size** |  |  |
| Ca_0.98_Eu_0.02_AlSiN_3_ | self-propagating high-temperature synthesis | ignition at 1050 °C, and further nitridation at 1450-1550 °C | atmospheric pressure | irregular particles | 6-9 µm | red phosphors | (Piao et al., 2007) |
| Mo_2_N | solid-state reaction | 700 ℃ | atmospheric pressure | nanoparticles |  | water splitting | (Chen et al., 2013) |
| 2D transition metal carbide/nitride heterostructure  nanosheets (h-TMCN) | high temperature solid state reaction | 800 °C、850 °C、900 °C | atmospheric pressure | nanosheets | ≈45 nm | water oxidation and full water splitting. | (Kou et al., 2019) |
| h-BN | CVD | 1040 °C | atmospheric pressure | thin films | film thickness <4 nm | single photon emitters | (Abidi et al., 2019) |
| CuNCo_3-x_V_x_ | solvothermal synthesis | 420 °C | atmospheric pressure | nanoparticles and nanowires | nanoparticle：50 nm | oxygen-evolution reaction electrocatalyst. | (Zhang et al., 2019) |
| Ni_3_Mo_3_N | solvothermal synthesis | calcination at 400 °C and ammonolysis at 800 °C | atmospheric pressure | nanorods | 60 nm | supercapacitor | (Kumar et al., 2020) |
| NiMoN@NiFeN | ammonolysis | 500 °C | atmospheric pressure | core-shell morphology of the nanoparticle-decorated nanorods | thickness of the NiFeN shell：100 nm | 3D core-shell OER catalyst for alkaline seawater splitting | (Yu et al., 2019) |
| Co_x_N/C | ammonolysis | 300 °C、360 °C、460 °C | atmospheric pressure | core-shell | oxygen shell：2 nm | ORR electrocatalysts in alkaline fuel cells | (Yang et al., 2019) |
| Ni_0.5_Co_2.5_N/NCNT | ammonolysis | 300 °C、400 °C、500 °C、 600 °C、 700 °C | atmospheric pressure | grass-like structure with multi-scaled porosity | 30-50 nm | electrocatalysis of oxygen in zinc-air batteries | (He et al., 2018) |
| VN | ammonolysis | 600 ℃ | atmospheric pressure | porous laminate | 15-80 μm | carbon-free hosts for high-loading sulfur cathodes in lithium−sulfur batteries | (Liu et al., 2020a) |
| Co-g-C_3_N_4_@rGO | molecular precursors method | 600 °C | atmospheric pressure | piled layers |  | fuel cells | (Liu et al., 2013) |
| ZrN | sol-gel methods | 800 ℃ | atmospheric pressure | nanoparticles | 45±16 nm | zinc–air battery | (Yuan et al., 2020) |
| N-Doped  Graphene | salt melt synthesis | 900 °C | atmospheric pressure | 3D porous |  | Zn–air batteries | (Cui et al., 2018) |
| MoN_1.2_  WN_1.5_  Mo_0.7_W_0.3_N_1.2_ | salt melt synthesis | 650 ℃  750 ℃  650 ℃ | atmospheric pressure | nanosheets | 0.6 nm  0.9 nm  0.6 nm | hydrogen evolution | (Jin et al., 2020) |
| MXene@CNF/Li | spin steaming technology | 60 ℃ | atmospheric pressure | film(can folded into diverse shapes) | ~25 μm | lithium metal composite anode | (Wang et al., 2020) |
| g-C_3_N_4_ | microwave reaction | 1285 K | atmospheric pressure | irregular morphology |  | hydrogen generation | (Guo et al., 2016) |
| Ti_2_N | acid-etching | 40 ℃ | atmospheric pressure | stacked layers |  | electrochemical energy storage materials | (Soundiraraju et al., 2017) |
| MoN | liquid exfoliation | 650 ℃ | 650 ℃ | nanosheets | nanosheets | electrocatalysts for hydrogen production | (Xie et al., 2014) |
| c-CN | MOFs-assisted synthesis | 850 ℃ | vacuum | bamboo-like nanofiber hollow structure | diameter: ~80 nm | Li–S batteries and supercapacitors | (Cai et al., 2020) |
| Ni-SN@C | unsaturated nitriding  process | 500 ℃ | atmospheric pressure | nanoparticles | 5-10 nm | hydrogen evolution from seawater | (Jin et al., 2021) |
| Fe_3_N_2_、FeN_2_、FeN_4_ | laser-heating | 1900 ±200 K 2100 ±200 K  >2000 K | 49.6 GPa、58.5GPa、135 GPa |  |  | HEDMs | (Bykov et al., 2018) |


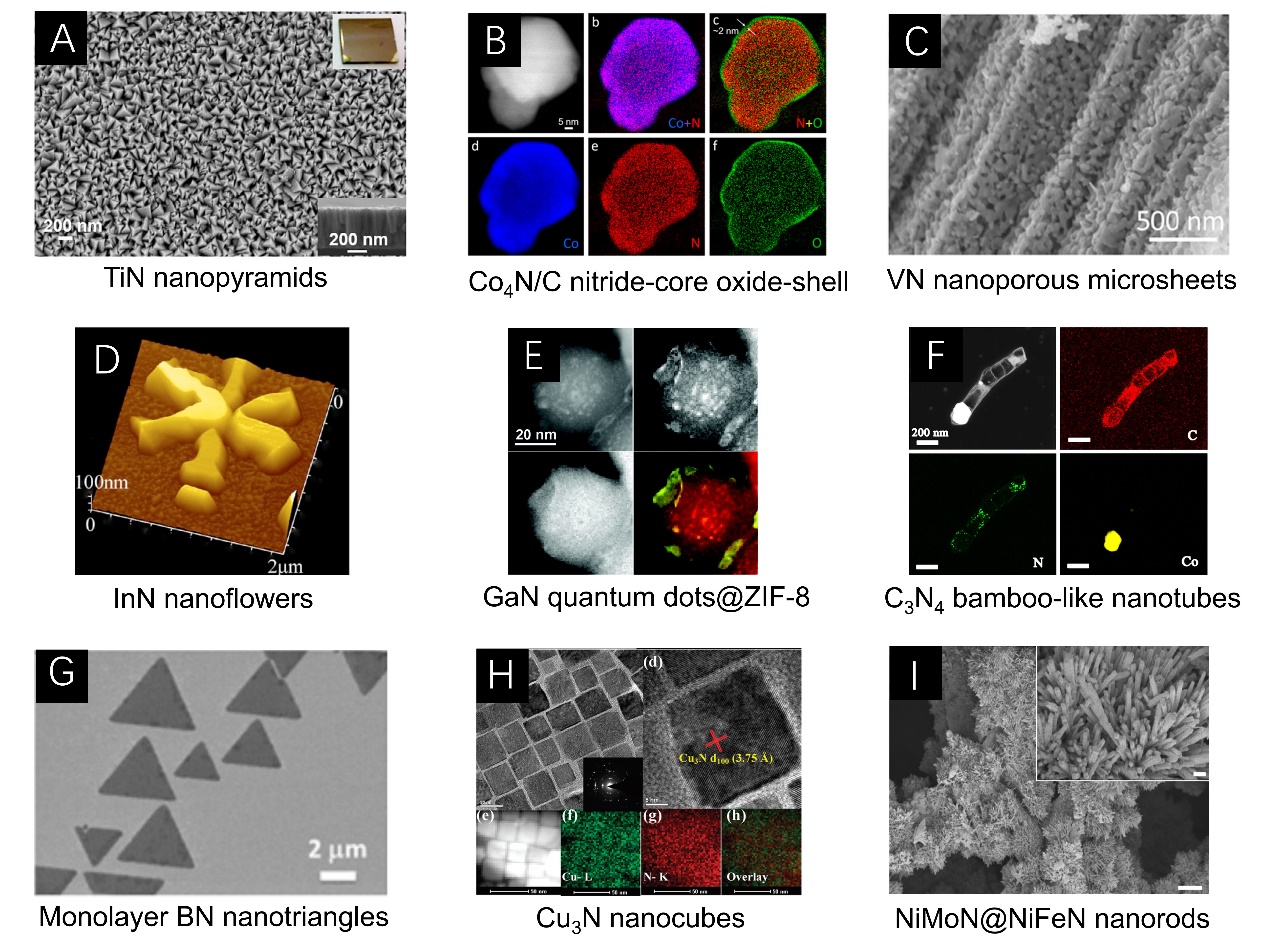


**Figure S1.** SEM/TEM/AFM images of inorganic nitride materials with various nanostructures: **(A)** TiN nanopyramids. **(B)** Co_4_N/C nitride-core oxide-shell. **(C)** VN nano-porous microsheets. **(D)** InN nano-flowers. **(E)** GaN quantum dots@ZIF. **(F)** C_3_N_4_ bamboo-like nano-tubes. **(G)** Monolayer BN nano-triangles. **(H)** Cu_3_N nano-cubes. **(I)** NiMoN@NiFeN nano-rods. Reproduced with permission from (Yu et al., 2019, Yang et al., 2019, Liu et al., 2020a, Arif et al., 2019, Kang et al., 2006, Esken et al., 2011, Liu et al., 2020b, Stehle et al., 2015, Wu et al., 2011).

Thin films of nano-pyramid TiN were sputtered on 304L steel substrate using DC magnetron reactive sputtering and had good electrochemical performances in symmetric full-cell supercapacitor. The Co_4_N/C nitride-core oxide-shell exhibited high ORR activity in alkaline fuel cells. The laminated stacking multiscale VN featuring interconnected holes possesses high storage space for sulfur loading, achieving high sulfur loading and utilization in lithium-sulfur batteries. The hexangular symmetry InN nanoflowers were prepared by MOCVD at atmospheric pressure. Very small GaN nanoparticles are caged in the sodalite-type pore structure ZIF-8. The bamboo-like g-C_3_N_4_ electrocatalyst show excellent performance of the as-designed electrocatalyst toward ORR outperforms the commercial Pt/C catalyst in alkaline solution. The monolayer BN nano-triangles were produced by CVD at atmospheric pressure. The Cu_3_N nano-cubes nanocrystals exhibit electrocatalytic activity toward oxygen reduction and appear to be promising cathodic electrocatalysts in alkaline fuel cells. The 3D core-shell NiMoN@NiFeN nano-rods are eminently active and durable oxygen evolution reaction catalyst for alkaline seawater electrolysis.


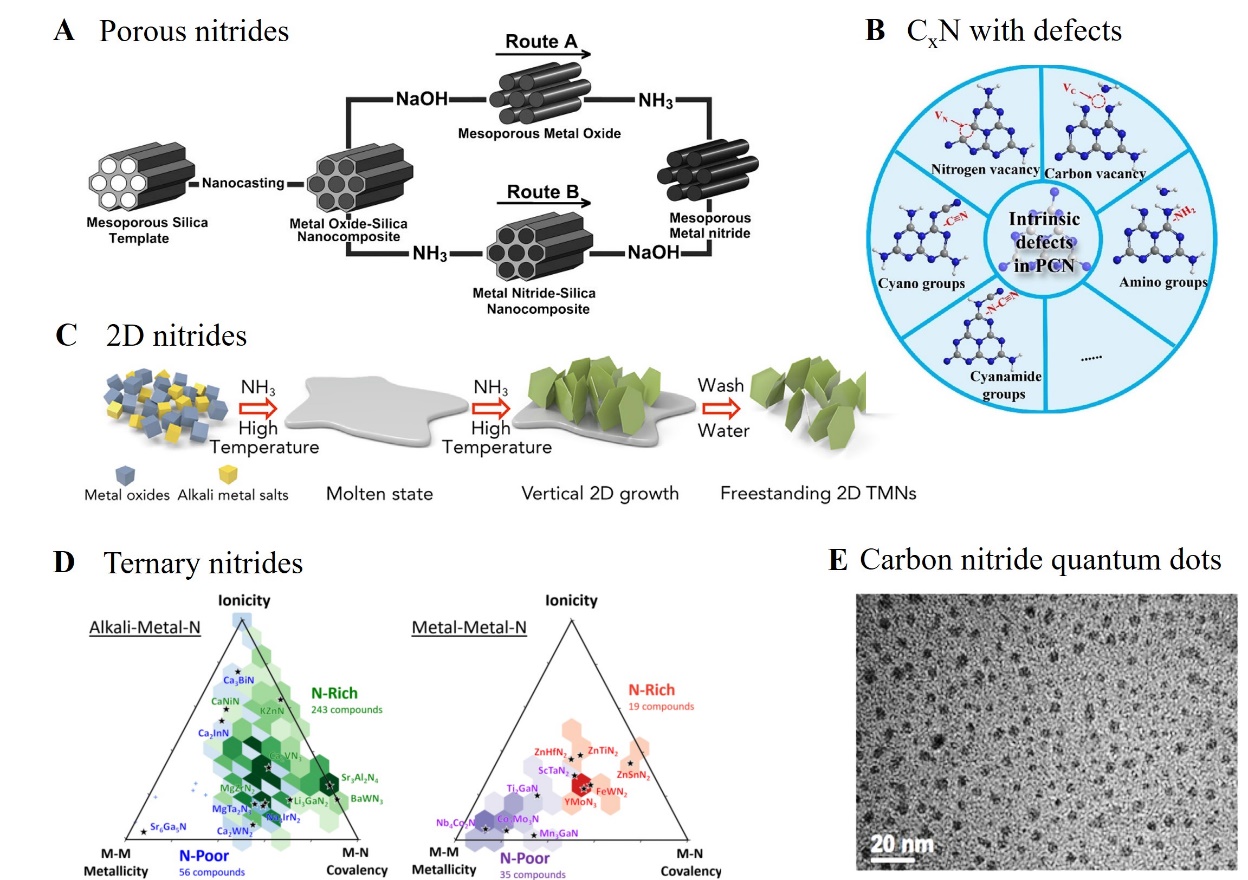


**Figure S2.** **(A)** The scheme of two pathways for the synthesis of self-supported mesoporous CoN and CrN. Reproduced with permission from (Shi et al., 2008). **(B)** Classifications of intrinsic defects in PCN photocatalysts. Reproduced with permission from (Meng et al., 2020). **(C)** Schematic of the synthesis process of 2D-MoN nanosheets. Reproduced with permission from (Jin et al., 2020). **(D)** Metallicity, ionicity, and covalency of the stable ternary nitrides; hexagonally binned on van Arkel triangles by the nitrogen-excess or nitrogen-deficiency of the ternary, compositionally-referenced against the deepest-hull binary nitrides. Reproduced with permission from (Sun et al., 2019). **(E)** The TEM image of carbon nitride quantum dots. Reproduced with permission from (Albolkany et al., 2020)

Reference

Piao, X., Machida, K. i., Horikawa, T., Hanzawa, H., Shimomura, Y.,Kijima, N. (2007). Preparation of CaAlSiN_3_: Eu^2+^ phosphors by the self-propagating high-temperature synthesis and their luminescent properties. *Chem. Mater. 19*, 4592-4599. doi: 10.1021/cm070623c

Chen, W. F., Iyer, S., Sasaki, K., Wang, C. H., Zhu, Y., et al. (2013). Biomass-derived electrocatalytic composites for hydrogen evolution. *Energ. Environ. Sci. 6*, 1818-1826. doi: 10.1039/c3ee40596f

Kou, Z., Wang, T., Gu, Q., Xiong, M., Zheng, L., Li, X., et al. (2019). Rational design of holey 2D nonlayered transition metal carbide/nitride heterostructure nanosheets for highly efficient water oxidation. *Adv. Energy Mater. 9*, 1803768. doi: 10.1002/aenm.201803768

Abidi, I. H., Mendelson, N., Tran, T. T., Tyagi, A., Zhuang, M., Weng, L. T., et al. (2019). Selective defect formation in hexagonal boron nitride. *Adv. Opt. Mater.* *7*, 1900397*.*  doi: 10.1002/adom.201900397

Zhang, J., Zhao, X., Du, L., Li, Y., Zhang, L., Liao, S., et al. (2019). Antiperovskite nitrides CuNCo_3-_*_x_*V*_x_*: highly efficient and durable electrocatalysts for the qxygen-evolution reaction. *Nano Lett. 19*, 7457-7463. doi: 10.1021/acs.nanolett.9b03168

Kumar, R., Bhuvana, T., Sharma, A. (2020). Ammonolysis synthesis of nickel molybdenum nitride nanostructures for high-performance asymmetric supercapacitors. *New J. Chem. 44*, 14067-14074. doi: 10.1039/d0nj01693d

Yu, L., Zhu, Q., Song, S., McElhenny, B., Wang, D., Wu, C., et al. (2019). Non-noble metal-nitride based electrocatalysts for high-performance alkaline seawater electrolysis. *Nat. Commun. 10*, 5106. doi: 10.1038/s41467-019-13092-7

Yang, Y., Zeng, R., Xiong, Y., Disalvo, F. J., Abruna, H. D. (2019). Cobalt-based nitride-core oxide-shell oxygen reduction electrocatalysts. *J. Am. Chem. Soc. 141*, 19241-19245. doi: 10.1021/jacs.9b10809

He, G., Han, X., Moss, B., Weng, Z., Gadipelli, S., Lai, F., et al. (2018). Solid solution nitride/carbon nanotube hybrids enhance electrocatalysis of oxygen in zinc-air batteries. *Energy Storage Mater. 15*, 380-387. doi: 10.1016/j.ensm.2018.08.020

Liu, R., Liu, W., Bu, Y., Yang, W., Wang, C., Priest, C., et al. (2020). Conductive porous laminated vanadium nitride as carbon-free hosts for high-loading sulfur cathodes in lithium-sulfur batteries. *ACS Nano 14*, 17308−17320. doi: 10.1021/acsnano.0c07415

Liu, Q., Zhang, J. (2013). Graphene supported Co-g-C_3_N_4_ as a novel metal-macrocyclic electrocatalyst for the oxygen reduction reaction in fuel cells. *Langmuir 29*, 3821-8. doi: 10.1021/la400003h

Yuan, Y., Wang, J., Adimi, S., Shen, H., Thomas, T., Ma, R., et al. (2020). Zirconium nitride catalysts surpass platinum for oxygen reduction. *Nat. Mater. 19*, 282-286. doi: 10.1038/s41563-019-0535-9

Cui, H., Jiao, M., Chen, Y. N., Guo, Y., Yang, L., Xie, Z., et al. (2018). Molten-salt-assisted Synthesis of 3D holey N-doped graphene as bifunctional electrocatalysts for rechargeable Zn-air batteries. *Small Methods 2*, 1800144. doi: 10.1002/smtd.201800144

Jin, H., Gu, Q., Chen, B., Tang, C., Zheng, Y., Zhang, H., et al. (2020). Molten salt-directed catalytic synthesis of 2D layered transition-metal nitrides for efficient hydrogen evolution. *Chem 6*, 2382-2394. doi: 10.1016/j.chempr.2020.06.037

Wang, C. Y., Zheng, Z. J., Feng, Y. Q., Ye, H., Cao, F. F.,Guo, Z. P. (2020). Topological design of ultrastrong MXene paper hosted Li enables ultrathin and fully flexible lithium metal batteries. *Nano Energy 74*, 104817. doi: 10.1016/j.nanoen.2020.104817

Guo, Y., Li, J., Yuan, Y., Li, L., Zhang, M., Zhou, C., et al. (2016). A rapid microwave-assisted thermolysis route to highly crystalline carbon nitrides forefficient hydrogen generation. *Angew. Chem. Int. Edit. 55*, 14693-14697. doi: 10.1002/anie.201608453

Cai, J., Song, Y., Chen, X., Sun, Z., Yi, Y., Sun, J., et al. (2020). MOF-derived conductive carbon nitrides for separator-modified Li-S batteries and flexible supercapacitors. *J. Mater. Chem. A 8*, 1757-1766. doi: 10.1039/c9ta11958b

Jin, H., Wang, X., Tang, C., Vasileff, A., Li, L., Slattery, A., et al. (2021). Stable and highly efficient hydrogen evolution from seawater enabled by an unsaturated nickel surface nitride. *Adv. Mater. 33*, 2007508. doi: 10.1002/adma.202007508

Bykov, M., Bykova, E., Aprilis, G., Glazyrin, K., Koemets, E., Chuvashova, I., et al. (2018). Fe-N system at high pressure reveals a compound featuring polymeric nitrogen chains. *Nat. Commun 9*, 2756. doi: 10.1038/s41467-018-05143-2

Arif, M., Sanger, A.,Singh, A. (2019). One-step sputtered titanium nitride nano-pyramid thin electrodes for symmetric supercapacitor device. *Mater. Lett. 245*, 142-146. doi: 10.1016/j.matlet.2019.02.082

Yang, Y., Zeng, R., Xiong, Y., DiSalvo, F. J., Abruna, H. D. (2019). Cobalt-based nitride-core oxide-shell oxygen reduction electrocatalysts. *J. Am. Chem. Soc. 141*, 19241-19245. doi: 10.1021/jacs.9b10809

Liu, R., Liu, W., Bu, Y., Yang, W., Wang, C., Priest, C., et al. (2020). Conductive porous laminated vanadium nitride as carbon-free hosts for high-loading sulfur cathodes in lithium-sulfur batteries. *ACS Nano 14,* 17308-17320. doi: 10.1021/acsnano.0c07415

Kang, T. T., Liu, X., Zhang, R. Q., Hu, W. G., Cong, G., Zhao, F. A., et al. (2006). InN nanoflowers grown by metal organic chemical vapor deposition. *Appl. Phys. Lett. 89*, 071113. doi: 10.1063/1.2337875

Esken, D., Turner, S., Wiktor, C., Kalidindi, S. B., Van Tendeloo, G.,Fischer, R. A. (2011). GaN@ZIF-8: Selective formation of gallium nitride quantum dots inside a zinc methylimidazolate framework. *J. Am. Chem. Soc. 133*, 16370-16373. doi: 10.1021/ja207077u

Liu, X., Yang, W., Chen, L., Liu, Z., Long, L., Wang, S., et al. (2020). Graphitic carbon nitride (g-C_3_N_4_)-derived bamboo-like carbon nanotubes/Co nanoparticles hybrids for highly efficient electrocatalytic oxygen reduction. *ACS Appl. Mater. Interfaces. 12*, 4463-4472. doi: 10.1021/acsami.9b18454

Stehle, Y., Meyer, H. M., Unocic, R. R., Kidder, M., Polizos, G., Datskos, P. G., et al. (2015). Synthesis of hexagonal boron nitride monolayer: control of nucleation and crystal morphology. *Chem. Mater. 27*, 8041-8047. doi: 10.1021/acs.chemmater.5b03607

Wu, H.,Chen, W. (2011). Copper nitride nanocubes: size-controlled synthesis and application as cathode catalyst in alkaline fuel cells. *J. Am. Chem. Soc. 133*, 15236. doi: 10.1021/ja204748u

Yu, L., Zhu, Q., Song, S., McElhenny, B., Wang, D., Wu, C., et al. (2019). Non-noble metal-nitride based electrocatalysts for high-performance alkaline seawater electrolysis. *Nat. Commun. 10*, 5106. doi: 10.1038/s41467-019-13092-7

Shi, Y., Wan, Y., Zhang, R., Zhao, D. (2008). Synthesis of self-Supported ordered mesoporous cobalt and chromium nitrides. *Adv. Funct. Mater. 18*, 2436-2443. doi: 10.1002/adfm.200800488

Meng, A., Teng, Z., Zhang, Q., Su, C. (2020). Intrinsic defects in polymeric carbon nitride for photocatalysis applications. *Chem. Asian J. 15*, 3405-3415. doi: 10.1002/asia.202000850

Jin, H., Gu, Q., Chen, B., Tang, C., Zheng, Y., Zhang, H., et al. (2020). Molten salt-directed catalytic synthesis of 2D layered transition-metal nitrides for efficient hydrogen evolution. *Chem. 6*, 2382-2394. doi: 10.1016/j.chempr.2020.06.037

Sun, W., Bartel, C. J., Arca, E., Bauers, S. R., Matthews, B., Orvañanos, B., et al. (2019). A map of the inorganic ternary metal nitrides. *Nat. Mater. 18*, 732-739. doi: 10.1038/s41563-019-0396-2

Albolkany, M. K., Wang, Y., Li, W., Arooj, S., Chen, C. H., Wu, N., et al. (2020). Dual-function HKUST-1: templating and catalyzing formation of graphitic carbon nitride quantum dots under mild conditions. *Angew. Chem. Int. Ed. Engl. 59*, 21499-21504. doi: 10.1002/anie.202009710
